# Are Anxiety Disorders in Children and Adolescents Less Impairing Than ADHD and Autism Spectrum Disorders? Associations with Child Quality of Life and Parental Stress and Psychopathology

**DOI:** 10.1007/s10578-017-0712-5

**Published:** 2017-02-07

**Authors:** Liesbeth G. E. Telman, Francisca J. A. van Steensel, Marija Maric, Susan M. Bögels

**Affiliations:** 1 Research Priority Area Yield, Research Institute of Child Development and Education, University of Amsterdam, Postbus 15780, 1001 NG Amsterdam, The Netherlands; 2 Department of Developmental Psychology, University of Amsterdam, Amsterdam, The Netherlands; 3 UvA minds, Academic Outpatient Child and Adolescent Treatment Center, Amsterdam, The Netherlands

**Keywords:** Anxiety disorders, Quality of life, Parental stress, Parental psychopathology

## Abstract

We compared clinically referred children with anxiety disorders (AD; *n* = 63) to children with autism spectrum disorder (ASD; *n* = 39), ADHD Combined (ADHD-C; *n* = 62), ADHD Predominantly Inattentive (ADHD-I; *n* = 64), and typically developing children (*n* = 42) on child quality of life (QOL), paternal and maternal psychopathology and parental stress. Diagnoses were based on DSM-IV-TR criteria. Multilevel analyses showed that QOL in AD was higher on school and social functioning, compared to respectively ADHD and ASD, and lower compared to normal controls on all five domains. Fathers reported their AD children higher QOL than mothers. Also, AD appeared to be associated with less parental stress and parental psychopathology than other child psychopathology. Therefore, parental factors may need to be considered more in treatment of children with ADHD/ASD than AD.

## Introduction

Anxiety disorders (AD) are by far the most prevalent mental disorders in childhood, with prevalence estimates ranging from 10 to 20% [1–3]. Interestingly, despite its prevalence, only 31% of AD children receive treatment [1]. Nevertheless, societal costs of childhood AD have been estimated high, with 9 to 20 times more costs than the normal population [4, 5]. Taking into account the high prevalence and societal costs, but low treatment usage, the question has raised whether AD might be less disturbing for the child and their parents than other common childhood mental disorders such as attention deficit hyperactivity disorder (ADHD) and autism spectrum disorder (ASD). To provide more insight into this issue, this study compared families of clinically referred children with AD to children with ADHD, ASD, and a non-clinical control group on child quality of life, parental psychopathology, and parental stress, in order to examine the functional impairment of childhood AD with respect to child and parental factors.

Currently, studies focusing on child psychiatric disorders have increasingly used quality of life as an assessment of functional impairment. The concept quality of life encompasses multiple domains, such as physical well-being, psychological well-being, and social functioning [6]. Comparisons of quality of life in various clinical groups have been scarce, even though it has been suggested that children who are diagnosed with a psychiatric disorder generally have a considerably poorer quality of life than children from the general population [6, 7]. Bastiaansen et al. [8] compared the quality of life between 310 clinically referred children referred to outpatient child psychiatric clinics with AD, ADHD, ASD, mood disorders, and other disorders, and showed that children with AD had a poorer quality of life in the domain of emotional functioning than children with ADHD, whereas the impact of AD on other aspects of quality of life was equal to children with ADHD, disruptive behavior disorders, and mood disorders [8]. Moreover, Thaulow and Jozefiak [9] found no significant differences between quality of life of clinically referred children with ADHD and children with anxiety/depression. Van Steensel et al. [10] found no differences between clinically referred children with ASD and comorbid AD and children with AD only in both parent and child reported quality of life; however, a lower quality of life was related to a higher anxiety severity and more severe autistic behavior. Thus, quality of life studies of clinically referred children with AD versus other mental disorders indicate that children with AD are at least as impaired as their counterparts with other disorders, but research is still sparse, and a comparison of several clinical groups with a control group seems to be missing.

Parental psychopathology has often been found to be elevated in parents of children with behavioral problems; however, it is unclear to what extent parents of children with AD have more or less psychopathology themselves than other parents, and how specific this relationship is. Moreover, likely heritability plays a role in the relationship between child and parental psychopathology, with heritability estimates of 30–40% for AD [11], and even stronger evidence for the heritability of ASD (> 90%, [12]) and ADHD (around 76% [13]). For AD, it has been found that (a) parental anxiety functions as a risk factor for child AD (e.g., [14]); (b) children with AD are more likely to have parents with AD as compared to other children [14, 15]; and (c) parental anxiety is related to child anxiety and depression, but not to child externalizing symptoms [16]. Moreover, a recent study showed that maternal anxiety symptoms were associated with all childhood problems, while paternal anxiety was only associated with childhood internalizing problems [17]. Research in other clinical groups found that parental depression was specifically associated with child internalizing problems, whereas parental ADHD was related to child psychopathology in general [18]. Thus far, different relations between child and parental psychopathology were found, and research is inconclusive whether these relations are specific or not and whether parents of one clinical group referred to mental health care appear to have more or less behavioral problems than other parents. Above all, most studies have examined the relation between child and parental psychopathology by measuring symptoms, instead of relying on childhood DSM-IV-TR disorders to define groups.

Parenting a child with a clinical diagnosis often raises parental stress, which can be defined as the distress that arises from the demands of parenting [19], and which may contribute independently to the clinical portrait of clinic-referred families [20]. The available evidence for differences in parental stress between children with different clinical diagnoses is scarce. The study of Costa et al. [20] showed that parental stress concerning dysfunctional interactions predicted child internalizing symptoms specifically; whereas parental stress concerning raising a difficult child predicted externalizing symptoms as well. In line, a comparison of children in a school-based system of care showed that parents of children with both high internalizing and externalizing symptoms reported more parental stress than children with low symptom levels [21]. Moreover, parents of children with internalizing problems reported comparable parental stress in parent–child interactions compared to parents of children with both internalizing and externalizing symptoms [21]. In addition, previous studies found that parents of children with ADHD combined subtype showed more parental stress than parents of ADHD inattentive subtype [22]. Finally, some studies have shown that parental stress was specifically associated with children’s anxiety symptoms [23]. Thus, both children’s internalizing and externalizing problems seem to be related to parental stress; however, little consideration has been given to the association between the types of child psychiatric diagnoses and parental stress. Knowledge about the role of parental stress in different child psychopathology is the first step to shed light on the role of parental stress in the etiology, maintenance, and treatment of child psychopathology.

The different roles of fathers and mothers in the development and maintenance of childhood AD have received more attention over the last decade [24]. Nevertheless, fathers still tend to be underrepresented in research [25]. However, fathers may be important informants as they may have a different perspective on their child’s problems (i.e., they tend to report less internalizing problems; [26]) and they may evaluate the burden of parenting a clinical child differently than mothers [27]. In addition, child internalizing problems have been found to be more closely related to internalizing symptoms of mothers than fathers [28]. Therefore, the current study included reports from both mothers and fathers, in order to examine possible differences between their reports.

In sum, research suggests that clinically referred children with AD are impaired in terms of quality of life; however, it is unclear whether this impairment is similar to other mental disorders. Parental impairment in children with AD was also found: parents of children with AD were more anxious themselves, and parents of children with internalizing problems experienced different parental stress than parents of children with externalizing problems. Parental impairment studies examined child anxiety symptoms instead of diagnosis and therefore no conclusions can be drawn about which childhood disorders are more or less associated with parental psychopathology or parental stress. The aim of this study was to investigate whether children with AD differ from children with different psychiatric disorders (i.e. ASD, ADHD) and a control group of children without a clinical diagnosis on a range of variables: quality of life of the child, parents’ internalizing and externalizing psychopathology, and parental stress, using both mother and father reports. Examining these possible differences is important because it may lead to further insight into the impact of childhood AD and may lead to the identification of possible treatment targets. Based on the above literature, we hypothesized that (1) children with AD would be at least as impaired in quality of life as children with ASD and ADHD [8, 9], and have a lower quality of life than children in the control group [7], (2) tentatively, parents of children with AD would have more internalizing and less externalizing problems than parents of children in the other clinical groups [14–16], and (3) parents of children with AD would likely experience similar levels of parental stress as parents of children in other clinical groups, but lower levels of stress compared to the control group [21].

## Method

### Participants

Participants eligible for this study were children and adolescents aged 6–21 years consecutively referred for clinical treatment to UvA minds, a community academic child mental health center in Amsterdam. Participants were included in this study when (1) children had a clinical diagnosis of AD, ASD, ADHD combined subtype (ADHD-C), or ADHD inattentive subtype (ADHD-I), (2) at least one of the parents had completed one of the questionnaires, and (3) had no comorbid clinical diagnosis. In addition, a control group of typically developing children without a clinical diagnosis was added. In total, 270 children (*M* = 11.70, *SD* = 2.92) were included, of which 264 (97.8%) mothers and 231 (85.6%) fathers participated (98.8% biological parents; 21.1% of the families were divorced). In 225 (83.3%) cases, both parents completed the questionnaires.

Of the 270 children, 63 (23.3%) children were diagnosed with AD, 39 (14.4%) with ASD, 62 (23.0%) with ADHD-C, 64 (23.7%) with ADHD-I, and 42 (15.6%) children in the control group. Table 1 displays the demographics of the five groups. Groups differed in child age (*F* (4, 265) = 4.44, *p* = 0.002) and gender (χ^2^ (4, *N* = 270) = 18.53, *p* = 0.001): children in the ASD and control group were significantly older than children in the ADHD-C group, and there were relatively more girls in the AD group than in the other groups. Groups did not differ in ethnicity (χ^2^ (20, *N* = 270) = 16.54, *p* = 0.683) or parental age (mothers: *F* (4, 259) = 1.694, *p* = 0.152; fathers: *F* (4, 226) = 0.743, *p* = 0.563). Fathers did not differ in educational background, but mothers did (*F* (4, 253) = 9.030, *p* < 0.001): mothers of the control group had a lower educational background than mothers of the other groups (*p* values < 0.001).

**Table 1:** Demographics of sample categorized by diagnosis

	Total (N = 270)		AD (*n* = 63)		ASD (*n* = 39)		ADHD-C (*n* = 62)		ADHD-I (*n* = 64)		Control (*n* = 42)	
Gender boys (*n, %*)	182	67.4	31	49.2	32	82.1	50	80.6	42	65.6	27	64.3
Age child (*M, SD*)	11.70	2.92	11.73	0.33	12.49	0.57	10.60	0.33	11.58	0.38	12.71	0.39
Age mother (*M, SD*)	44.95	5.15	45.73	0.63	45.10	0.76	43.67	0.70	45.63	0.71	44.44	0.68
Age father (*M, SD*)	47.03	5.70	47.48	0.75	47.09	0.85	45.96	0.79	47.68	0.85	46.83	0.93
Ethnic background (*n*, % Dutch)	207	76.7	45	71.4	30	76.9	46	74.2	47	73.4	39	92.9
Educational level mother^a^ (*M, SD*)	5.56	1.34	5.63	0.17	6.08	0.15	5.69	0.16	5.68	0.18	4.41	0.19
Educational level father^a^ (*M, SD*)	5.59	1.45	5.48	0.23	5.91	0.19	5.90	0.18	5.39	0.23	5.26	0.22

^a^Range 1–7: 1 = Primary school, 7 = University

### Procedure

**Fig. 1:**
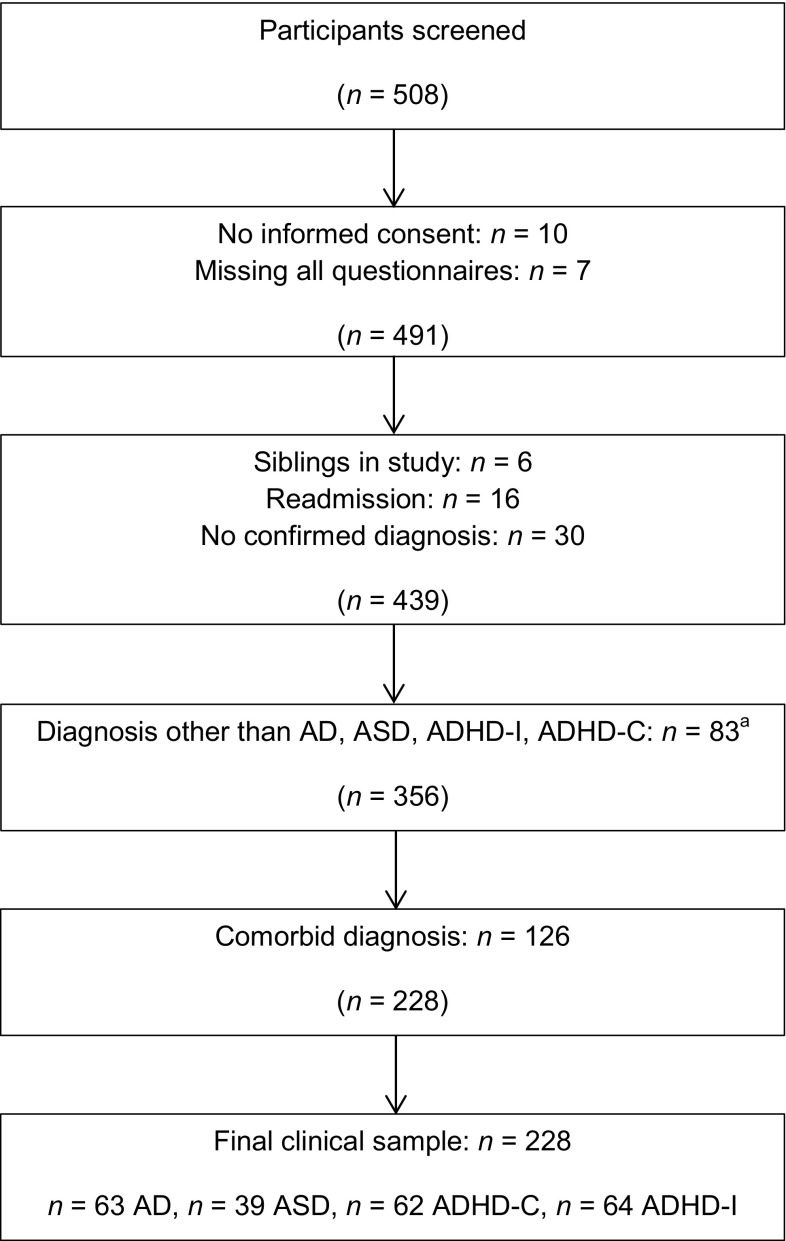
Flow chart of recruitment process clinical groups. ^a^Other diagnoses included: ADHD not otherwise specified (*n* = 23), ADHD predominantly hyperactive subtype (*n* = 7); oppositional defiant disorder (*n* = 5); mood disorders (*n* = 10); other disorder of infancy, childhood, or adolescence (*n* = 26); adjustment disorder (*n* = 2); other disorder (*n* = 10), including: body dysmorphic disorder (*n* = 1*)*; dissociative disorder (*n* = 1), learning disorder not otherwise specified (*n* = 2), eating disorder (*n* = 1), tic disorder not otherwise specified (*n* = 4), undifferentiated somatoform disorder (*n* = 1)

Data was collected from May 2012 to May 2014 using online questionnaires that are part of the intake procedure of the treatment center. All participants were informed about the study, and ethical approval for the study as well as informed consent was obtained. Only children with an IQ > 70 are admitted at the treatment center. Children were excluded when (a) their parents did not give informed consent; (b) they were re-admitted and already participated in the study; (c) one of their siblings participated in the study; (d) diagnosis was not (yet) confirmed; (e) they had a primary diagnosis other than AD, ASD, ADHD-C, or ADHD-I. Figure 1 shows the flow chart of the recruitment process. Moreover, when more than two parents were involved, only data from the biological parents was used. 356 children were eligible for this study based on their primary diagnosis of AD, ASD, ADHD-C, or ADHD-I. It was chosen to only include children without comorbid diagnoses because we wanted to be able to tease apart the possible differences between the disorders and the variables of interest (quality of life, parental psychopathology and parental stress). That is, if we allowed comorbidity in the groups, we could no longer make inferences about children with a particular disorder having a lower quality of life, or parents of children with a particular disorder having higher levels of stress or psychopathology, which was the main aim of this study. Therefore, 126 (35.6%) children were excluded from analyses (38 AD, 29 ASD, 33 ADHD-C, 26 ADHD-I) because they had a comorbid diagnosis (AD, ADHD, ASD, mood disorders or oppositional defiant disorders). The excluded group did not differ from the included group in age, gender, parental psychopathology or parental stress. The groups did differ in terms of child psychopathology as reported by the father (included group had less total and internalizing problems on the CBCL, resp. *t*(292) = 2.32, *p* = 0.021; *t*(293) = 3.02, *p* = 0.003), and psychological well-being as reported by the father (included group had better psychological well-being, *t*(285) = −2.24, *p* = 0.026), but did not differ on any of the measures according to mother report (*p-*values > 0.10). Of the included children, 4.8% used medication at time of intake (methylphenidate, atomoxetine or dexamphetamine, used by 5.2% of the children with ASD; 4.8% ADHD-C; 9.4% ADHD-I), compared to 16.7% of the excluded children.

DSM-IV-TR diagnoses were made by a multidisciplinary team within the treatment center and were based on diagnostic assessments (including for example records on developmental history, observations, diagnostic interviews and psychological and/or psychiatric evaluations). In addition, a standardized interview was administered in nearly half of the cases (n = 103); i.e. the ADIS-C/P [29] for anxiety disorders and ADHD, and the ADOS [30] and/or ADI-R [31] for ASD, that confirmed the diagnosis of the multidisciplinary team. In line with the clinical classification of subtypes of ADHD as defined in the DSM-IV-TR and DSM-5 [32, 33], the ADHD group was divided into a group with children with both hyperactivity and inattention problems (ADHD-C) and a group with predominantly inattention problems (ADHD-I). Due to small sample size, children with ADHD predominantly hyperactive/impulsive type were excluded from the study.

Next to the clinical diagnosis assessment, we compared clinical groups on the Child Behaviour Checklist (CBCL) [34] to confirm their diagnosis by clinical profile. Differences on the CBCL subscales were examined using the same technique as the analyses described in the “Statistical analyses” section. Table 2 shows the means on the different subscales. Children with AD had more anxiety problems and withdrawn behavior than children with ADHD and control children. Children with ASD showed more withdrawn behavior and social problems than AD children, while children with ADHD (and ASD) showed more attention problems as well as more externalizing behaviors (rule-breaking and aggressive behaviors) than AD children. Children in the control group were found to have lower scores than each of the clinical groups on every subscale. These findings support the (DSM-IV-TR-based) distinction that is made in this sample between the four clinical groups and the control group.

**Table 2:** Means (M) and standard deviations (SD) of scores on CBCL and KIDSCREEN, classified by diagnosis

	AD	ASD	ADHD-C	ADHD-I	Control
	*M (SD)*	*M (SD)*	*M (SD)*	*M (SD)*	*M (SD)*
CBCL					
Anxious	9.03 (5.25)	7.93 (4.42)	5.18 (3.61)^b^	4.85 (3.83)^b^	1.44 (2.32)^b^
Withdrawn	4.35 (3.29)	7.07 (3.56)^a^	2.28 (2.05)^b^	3.37 (2.57)^b^	1.18 (1.53)^b^
Somatic	4.25 (3.29)	3.60 (3.08)	2.43 (2.41)^b^	3.17 (3.02)	0.97 (1.40)^b^
Social	4.51 (3.62)	7.16 (3.87)^a^	5.35 (3.17)	3.80 (3.18)	0.96 (1.38)^b^
Thought	5.39 (4.26)	6.41 (3.70)	4.54 (3.05)	4.19 (3.34)	1.24 (1.83)^b^
Attention	5.58 (3.89)	8.70 (3.78)^a^	11.20 (2.59)^a^	10.75 (3.73)^a^	3.58 (3.13)^b^
Rule-breaking	2.02 (1.92)	3.82 (3.13)^a^	4.64 (3.10)^a^	2.81 (2.60)^a^	0.85 (1.15)^b^
Aggressive	6.30 (5.77)	10.27 (6.29)^a^	12.56 (5.48)^a^	7.00 (5.59)	2.42 (2.76)^b^
KIDSCREEN					
Physical well-being	49.41 (12.94)	46.52 (11.30)	54.87 (11.12)	51.13 (11.75)	57.77 (11.48)^a^
Psychological well-being	44.25 (11.77)	39.76 (9.69)	46.21 (10.03)	46.87 (7.24)	54.08 (9.16)^a^
Autonomy and parent child relations	52.57 (9.32)	50.40 (8.55)	50.46 (8.54)	50.52 (6.74)	56.24 (9.13)^a^
Social support and peers	50.64 (11.44)	43.34 (11.47)^b^	51.49 (8.44)	53.70 (8.70)^a^	55.66 (6.76)^a^
School environment	49.57 (10.57)	45.14 (9.25)	43.01 (8.54)^b^	43.01 (7.47)^b^	54.15 (9.13)^a^

Groups are compared to the reference group of AD children. Subscripts indicate which group differs from the AD group

^a^Group has significantly higher score than AD

^b^Group has significantly lower score than AD

Typically developing children were recruited by graduate students via schools, day care facilities, and convenience sampling. Children were excluded from the control group when their parents reported that they had a psychiatric diagnosis (DSM-IV-TR; [32]), or when they received (or had received) support or treatment from a child mental health care center in the past year. Low scores on the CBCL of the control group, comparable to those found in the general population [35], further supported the representativeness of the control sample.

### Instruments

#### Quality of Life

Quality of life was measured with the KIDSCREEN-27 quality of life questionnaire [36]. This study used the proxy report, designed for parents. The KIDSCREEN-27 measures five dimensions: Physical Well-being (5 items); Psychological Well-being (7 items); Autonomy and Parent Child Relations (7 items); Social Support and Peers (4 items); and School Environment (4 items). An example item is: ‘Did your child have the chance to talk to his/her parents when he/she wanted to?’ (item of the Autonomy and Parent Child Relations scale). The items are scored on a 5-point scale ranging from 1 = *never*/*not at all* to 5 = *always*. Internal consistency values in this study range between 0.73–0.92 for mother reports, and between 0.78–0.89 for father reports.

#### Parental Psychopathology

Parental psychopathology was assessed with the Adult Self Report (ASR) [37]. The ASR measures a broad range of behavioral problems in adults and contains 123 items which are rated on a 3-point scale (0 = *not at all*, 1 = *sometimes*, 2 = *often*). An example item is: ‘I cannot get along with other people’. A total problem scale is created by summing the score of all items. This study also used the two broad-band syndrome scales: internalizing and externalizing problems. Internal consistency values in this study range between 0.85–0.96 for mother reports and between 0.84–0.95 for father reports. Means and standard deviations are shown in Table 3.

**Table 3:** Means (M) and standard deviations (SD) of scores on ASR and NOSI, classified by diagnosis and respondent

	AD		ASD		ADHD-C		ADHD-I		Control	
	M	F	M	F	M	F	M	F	M	F
	*M (SD)*	*M (SD)*	*M (SD)*	*M (SD)*	*M* (SD)	*M* (SD)	*M (SD)*	*M (SD)*	*M (SD)*	*M (SD)*
ASR										
Total	33.74 (25.14)	28.54 (25.08)	28.97 (17.47)	32.85 (17.19)	37.39 (20.02)	32.87 (22.20)	42.37 (24.99)^a^	33.35 (17.92)^a^	22.78 (20.61)^b^	13.73 (14.10)^b^
Internalizing	12.24 (10.42)	10.10 (10.60)	9.28 (6.79)	9.45 (7.14)	11.93 (7.48)	9.33 (8.71)	14.24 (9.15)	9.35 (7.23)	7.87 (8.36)^b^	3.36 (4.58)^b^
Externalizing	7.11 (5.93)	6.04 (5.76)	6.36 (4.98)	9.06 (6.73)	9.03 (5.80)	8.54 (6.10)	8.65 (6.91)	9.33 (5.06)	4.26 (4.22)^b^	3.29 (3.14)^b^
NOSI										
Stress	54.66 (23.28)	53.22 (23.60)	72.71 (22.96)^a^	75.63 (25.85)^a^	74.10 (21.22)^a^	64.46 (22.66)^a^	59.18 (21.04)	51.82 (19.71)	43.54 (19.48)	39.87 (17.74)

Groups are compared to the reference group of AD children. Subscripts indicate which group differs from the AD group

*M* Mother, *F* Father

^a^Group has significantly higher score than AD

^b^Group has significantly lower score than AD

#### Parental Stress

Parental stress was assessed with the Nijmeegse Ouderlijke Stress Index Kort (NOSI-K) [38], a short version of the Dutch version of the Parenting Stress Index (PSI). The NOSI-K consists of 25 items, which are rated on a six-point scale, ranging from 1 = *I completely disagree* to 6 = *I completely agree*. The NOSI-K is a short version of the NOSI (123 items), and is constructed by combining the 25 items with the highest factor loadings on the scale ‘general parenting stress’. An example item: is ‘My child is more demanding to me than most other children’. Internal consistency values in this study are 0.94 (mothers) and 0.95 (fathers). Means and standard deviations are shown in Table 3.

### Statistical Analyses

Multilevel analyses using maximum likelihood estimation procedures were used to examine group differences in child quality of life, parental psychopathology, and parental stress. Multilevel data analysis is used when data is nested, in this case mothers and fathers reported about the same child. Their reports were treated as repeated measures. An advantage of multilevel data analysis is that it does not require complete data over measures, nor is there a need for equal numbers of cases in each group [39]. Therefore, all available data was used in analysis, including those cases of which one parent did not participate. Moreover, multilevel analysis takes into account dependency among respondents, as both respondents in this study are nested within one child (grouping variable).

Each (sub)scale of the different measures (KIDSCREEN-27, ASR, NOSI-K) was used separately as a dependent variable in analyses. Prior to analysis, several univariate outliers were detected for each dependent variable (−3.29 > z > 3.29, *p* < 0.001). Outliers were trimmed to acceptable z-scores, and analyses were run with the original and adjusted data. There were no differences in outcomes; therefore, it was decided to report results of the analyses including outliers. No multivariate outliers were detected using Mahalonobis distance at α = 0.001. Assumptions were checked for the residuals of the different models. Not all variables approached a normal distribution; however, transformations did not improve normality thus the original distributions were used.

Clinical groups were treated as dichotomous measures, by creating dummy variables, and the dummy variables of ASD, ADHD-C, ADHD-I, and the control group were used as predictors. The reference group in each analysis is the group of children with AD. The continuous variables were transformed into standardized scores. In this way the parameter estimates of the dummy variables can be interpreted as a measure of effect size (Cohen’s *d*). Child age, child gender, and maternal education were added as covariates in analyses, in order to account for existing differences between the groups. Respondent (father vs. mother) was added as a predictor as well, and interactions between respondent and clinical group were added to the model. When a significant interaction was found, additional analyses were run within the AD group and within the concerning other group with respondent as a single predictor, in order to interpret the interaction effect.

## Results

### Quality of Life

Results indicated that (a) children with AD had a higher quality of life in school functioning compared to children with both types of ADHD, (b) children with AD had a higher quality of life in social functioning than children with ASD, but had a lower social functioning than children with ADHD-I, and (c) no significant differences between AD and the other clinical groups on the other three domains (physical well-being, psychological well-being, and autonomy and parent child relations). In addition, children in the control group were reported to have a higher quality of life than all other children on all five dimensions except on the subscale social functioning where children in the control group did not differ from children with ADHD-I.

A significant interaction effect between respondent and ASD (compared to reference group AD) was found for the two domains psychological well-being and autonomy and parent–child relation, showing that fathers reported a higher quality of life for children with AD than mothers (resp. *β* = 0.39, *p* = 0.006 and *β* = 0.41, *p* = 0.015), but mothers and fathers reported comparable scores for children with ASD. Table 4 shows the parameter estimates, which indicate differences of the groups compared to the AD group: a positive parameter indicates a higher quality of life than the AD group, while a negative parameter indicates a lower quality of life than AD children.

**Table 4:** Parameter estimates of the models concerning the KIDSCREEN quality of life dimensions

Predictors	PHY	PSY	AUT	SOC	SCH
	*β (SE)*	*β (SE)*	*β (SE)*	*β (SE)*	*β (SE)*
Intercept	1.07** (0.31)	−0.49 (0.33)	0.45 (0.32)	0.19 (0.33)	0.83* (0.33)
ASD	−0.22 (0.19)	−0.20 (0.19)	−0.07 (0.19)	−0.74** (0.20)	−0.29 (0.19)
ADHD-C	0.13 (0.16)	0.28 (0.17)	−0.17 (0.16)	−0.03 (0.18)	−0.64** (0.16)
ADHD-I	0.11 (0.16)	0.30 (0.16)	−0.18 (0.16)	0.39* (0.18)	−0.62** (0.16)
Control	0.84** (0.20)	1.05** (0.20)	0.70** (0.20)	0.57** (0.22)	0.66** (0.20)
RES	−0.00 (0.12)	0.38** (0.11)	0.39* (0.15)	0.00 (0.12)	0.14 (0.12)
AGE	−0.09** (0.02)	−0.02 (0.02)	−0.03 (0.02)	0.00 (0.02)	−0.05** (0.02)
Gender	−0.49** (0.11)	−0.24* (0.12)	−0.26* (0.11)	−0.27* (0.12)	0.16 (0.12)
MEDU	−0.01 (0.04)	−0.11* (0.04)	−0.01 (0.04)	−0.03 (0.04)	−0.02 (0.04)
ASD*RES	−0.21 (0.19)	−0.46** (0.18)	−0.50* (0.24)	−0.05 (0.20)	−0.12 (0.18)
ADHD-C*RES	0.24 (0.17)	−0.28 (0.16)	−0.32 (0.22)	0.15 (0.18)	0.02 (0.16)
ADHD-I*RES	−0.05 (0.17)	−0.14 (0.16)	−0.17 (0.21)	−0.23 (0.17)	0.01 (0.16)
Control*RES	−0.04 (0.20)	−0.51** (0.19)	−0.33 (0.26)	−0.17 (0.21)	−0.18 (0.19)

*PHY* physical well-being, *PSY* psychological well-being, *AUT* autonomy and parent child relations, *SOC* social support and peers, *SCH* school environment, *RES* respondent: 0 = mother, 1 = father; *Gender* 0 = boy, 1 = girl, *MEDU* maternal education

*p < 0.05; **p < 0.01

### Parental Psychopathology and Parental Stress

No differences in total parental psychopathology were found between parents of children with AD and parents of children with ASD and ADHD-C. However, parents of children with AD reported less psychopathology than parents of children with ADHD-I, and reported more psychopathology than parents of control children.

With respect to specific forms of parental psychopathology, parents of children with AD reported similar levels of internalizing and externalizing problems as parents of children with ASD and both types of ADHD. Furthermore, a significant interaction effect between respondent and ASD was found; additional analyses showed that fathers of children with ASD showed borderline significantly more externalizing problems than mothers (*β* = 0.46, *p* = 0.061), whereas fathers and mothers of children with AD reported similar amounts of problems.

Parents of children with AD reported lower parental stress than parents of children with ADHD-C and parents of children with ASD. No differences in parental stress were found between parents of children with AD, ADHD-I and normal control children. Finally, a significant interaction effect between respondent and ADHD-C was found; additional analyses showed that fathers of children with ADHD-C showed less parental stress than mothers (*β* = −0.40, *p* = 0.009), whereas fathers and mothers of children with AD reported similar amounts of parental stress. Parameter estimates are shown in Table 5, positive parameters indicate higher levels of parental psychopathology and stress compared to parents of the reference group of AD children, and negative parameters indicate lower levels of parental psychopathology and stress than parents of AD children.

**Table 5:** Parameter estimates of the models concerning ASR total problems, internalizing, and externalizing problems, and NOSI parental stress

Predictors	TOTAL	INT	EXT	STRESS
	*β (SE)*	*β (SE)*	*β (SE)*	*β (SE)*
Intercept	−0.19 (0.32)	0.36 (0.33)	−0.15 (0.30)	−0.77* (0.31)
ASD	−0.16 (0.21)	−0.28 (0.20)	−0.11 (0.20)	0.77** (0.18)
ADHD-C	0.22 (0.18)	0.03 (0.18)	0.34 (0.18)	0.84** (0.16)
ADHD-I	0.40* (0.18)	0.25 (0.18)	0.27 (0.17)	0.18 (0.16)
Control	−0.56* (0.23)	−0.57** (0.22)	−0.53* (0.22)	−0.25 (0.20)
RES	−0.18 (0.16)	−0.18 (0.15)	−0.16 (0.17)	−0.06 (0.13)
Age	0.01 (0.02)	0.02 (0.02)	0.01 (0.02)	−0.02 (0.02)
Gender	0.12 (0.11)	0.12 (0.11)	0.02 (0.10)	0.14 (0.11)
MEDU	−0.06 (0.04)	−0.07 (0.04)	−0.05 (0.04)	0.12** (0.04)
ASD*RES	0.40 (0.25)	0.25 (0.23)	0.62* (0.28)	0.15 (0.20)
ADHD-C*RES	0.00 (0.22)	−0.09 (0.21)	0.09 (0.25)	−0.36* (0.18)
ADHD-I*RES	−0.21 (0.22)	−0.37 (0.20)	0.27 (0.24)	−0.20 (0.18)
Control*RES	−0.16 (0.27)	−0.31 (0.25)	0.02 (0.29)	−0.09 (0.22)

*TOTAL* total problems, *INT* Internalizing problems, *EXT* Externalizing problems, *STRESS* parental stress, *RES* respondent: 0 = mother, 1 = father, *Gender* 0 = boy, 1 = girl, *MEDU* maternal education

*p < 0.05; **p < 0.01

## Discussion

The goal of this study was to compare families of children with AD to families of children with ASD, ADHD-C, ADHD-I, and a control group on child quality of life, parental psychopathology and parental stress. Most important findings can be summarized as follows: (a) Children with AD had a lower quality of life than typically developing children, a higher quality of life than children with ASD on the domain social functioning, and a higher quality of life than children with both types of ADHD on the domain school functioning, but did not differ from the other clinical groups on physical well-being, psychological well-being and autonomy and parent–child relation; in addition, fathers of AD children reported higher quality of life than mothers on the domains psychological well-being and autonomy and parent–child relation; (b) parents of children with AD had less psychopathology than parents of children with ADHD-I, but more than parents of typically developing children, and did not differ in psychopathology from parents of children with ADHD-C and ASD; (c) no evidence for specificity was found: parents of children with AD reported similar levels of internalizing and externalizing problems as parents of children with ASD and both types of ADHD; (d) parents of children with AD experienced less parental stress than parents of children with ASD and ADHD-C, but did not differ in parental stress from parents with ADHD-I and parents of the control group; and (e) mothers and fathers of AD children reported comparable levels of parental stress, in contrast to fathers of ADHD-C children who reported less parental stress than mothers.

First, this study showed that clinically referred children with AD are as impaired in parent-reported quality of life as children with other clinical diagnoses in three out of five domains. However, for two domains a significant difference was found, which contrasts the findings of [8, 9] that AD children do not seem to differ from children with ADHD, except for emotional functioning [8]. This may have to do with the lack of comorbid disorders in the current sample and/or the different instrument used to measure quality of life. Although not reflected in quality of life, these studies did find lower academic performance in ADHD compared to AD [8, 9], which could explain the difference in school functioning between these groups that was found in the current study. More specific, we found that children with AD had higher scores with respect to their school functioning than children with both types of ADHD and children with ASD. It is interesting that problems of children with AD seem to interfere less with their school functioning when compared to other clinical groups (however, note that the control group had a significantly higher quality of life in the domain of school functioning than children with AD). This is in line with the finding of Weitkamp et al. [40] that externalizing, but not internalizing, psychopathology was associated with low functioning at school. Moreover, our findings are also in line with the finding that children with AD are found to be impaired in school functioning, although children with a comorbid externalizing disorder show the greatest impairment [41]. In addition, children with AD had a better social functioning than children with ASD, which is in line with the clinical presentation of ASD [33] and the finding that children with ASD have a poorer quality of life than children with other clinical diagnoses [8]. Concluding, AD children are impaired in quality of life compared to normal controls, but show fewer impairments in school and social functioning than children with ADHD-C and ASD respectively. It may be that the severity of the disorder (or the severity of particular symptoms of the disorder) plays a role in how well children function in each quality of life domain. For example, severity of attention problems (especially seen in ADHD; [42]) may account for larger impairments in school, while more social skills problems (often seen in ADHD and ASD; [43]) may be related to more social functioning impairments. Indeed, severity of anxiety and autistic problems were related to a lower quality of life in the study of Van Steensel et al. [10]. In this respect, it may be interesting for future studies to measure disorder severity and/or symptom severity and investigate how it relates to each domain of quality of life.

Although no differences between the AD group and the other clinical groups were found, an interesting finding was that fathers of children with AD reported a higher quality of life compared to mothers on both psychological well-being and autonomy and parent relations. Earlier research into informant agreement on quality of life among children with different psychiatric disorders using the KIDSCREEN-27 found moderate correlations between father-mother agreement, but did not find fathers and mothers to differ in their child reports [44]. However, they did not examine agreement for each separate clinical group. It is possible that the same mechanism for the difference in reports of internalizing problems is underlying the difference in quality of life, that is, mothers spending more time with their child and thus being more aware of internalizing problems [45]. Alternatively, father involvement in raising children has increased and it could also be that fathers’ perception of their child differs from mothers’ perception [25, 46]. Tentatively, viewing the child as less impaired may serve fathers’ role of helping their child overcome anxiety by challenging the child (e.g., [47]). That is, if fathers would view their child as highly impaired they would have more difficulty challenging the child to explore new territory and cross their own borders. However, more research into the (different) perspective of mothers and fathers regarding their child’s quality of life and whether it is dependent on the child’s clinical diagnosis is necessary before firm conclusions can be drawn.

With respect to parental psychopathology, this study does not support a specific link between child and parental internalizing problems as parents of children with AD reported no elevated levels of internalizing problems themselves compared to parents of the other clinical groups, which is in contrast with previous studies [15], but in line with a recent study that found little evidence of specificity in offspring mental disorders [48]. This study also found that parents of children with AD experience more psychopathology themselves than parents of typically developing children, suggesting that child and parental psychopathology are related. Moreover, no differences were found in parents own psychopathology reports between fathers and mothers from each clinical group. This is in line with earlier research [17], and underlines the importance of both fathers’ and mothers’ contribution to child psychopathology and/or the child’s psychopathology affecting both fathers and mothers equally in terms of their own psychopathology.

This study found that levels of parental stress are lower among parents of children with AD as compared to ASD and ADHD-C. In addition, parents of AD children did not differ in parental stress from parents of children with ADHD-I and the control group. Thus, in contrast to previous findings [21], it seems that the externalizing behavior of children with ASD and ADHD-C is more associated with parental stress than the internalizing behavior of the children with AD. Research to date indicated that maternal stress (paternal stress was not investigated) is associated with children’s both anxious and depressive symptoms [23] as well as externalizing problems [49], but does not provide enough evidence for differences in parental stress between internalizing and externalizing disorders. However, it is possible that stress levels experienced by parents may include different domains; e.g., parenting a child with internalizing problems may lead to parental stress concerning parent–child interactions, while parenting a child with externalizing problems may lead to parental stress concerning raising a difficult child [21]. Finally, this study did not find differences between both parents’ reports regarding parental stress, which implies that fathers experience equal levels of stress as mothers while raising children with psychopathology, and thus suffer as much, and/or affect their children as much with their stress.

Strengths of this study were that (a) this study was one of the first to estimate the impact of childhood AD associated with child and parental factors, in comparison to other diagnostic groups and a control group of children without a clinical diagnosis, (b) reports from both mothers and fathers on each of the measures were included, (c) this study included clinically referred children and thus groups were based on DSM-IV-TR diagnoses instead of symptomatology, and (d) multilevel data analysis was used to address the dependent structure of the data. Limitations of this study also need to be addressed. First, as parents were the sole reporters about their child’s quality of life, their own emotional-behavioral problems and their levels of stress, it might be that the relations between these variables are overestimated because of distortion: parents’ own symptoms (and/or parental concern) influence their reports of their child’s behavior and can account for discrepancies between parents’ reports [45]. Moreover, the lack of an independent measure of quality of life potentially biased this study’s results, because results are now solely based on parental perception of the child’s quality of life. Future studies should include objective quality of life indicators in order to account for this bias. In addition, the difference in recruitment procedures between the clinical and control group potentially biased the current results; i.e., it is possible that the control group had a higher quality of life because they were not treatment-seekers. Next, this study included children with a single diagnosis, in order to avoid measuring shared symptoms due to comorbidity and to be able to state which diagnoses differed from each other in terms of quality of life, parental psychopathology, and parental stress. Nevertheless, differences in CBCL scores still showed comorbidity on a symptom level, indicating overlap in symptoms between clinical diagnoses. Moreover, comorbidity on a diagnosis level is frequently present in clinical practice, and excluding this group makes the generalizability of our findings to routine clinical practice limited. At the same time, note that (1) comorbidity in symptoms is different from comorbidity in diagnosis (e.g., not impairing for daily functioning, no target for treatment), (2) the CBCL scores for the different clinical groups followed the expected clinical profile (see Table 2), indicating valid differences in symptom profiles between diagnoses, and (3) the excluded comorbid group did not differ in (mother-reported) child psychopathology, quality of life, parental stress and psychopathology. Finally, considering the cross-sectional design of this study, no conclusions can be drawn on the direction of the association between child psychiatric diagnosis and parents’ problems. Therefore, it is not possible to disentangle to what extent higher reports of behavioral problems among parents of the clinical groups reflect the burden of parenting a child with a psychiatric disorder, or to what extent more psychopathology in parents causes more child problems in general. Likely, the relation is bidirectional: parents with behavior problems influence their child’s behavior, next to heritability [11], and children with behavioral problems influence parents’ behavior problems and stress; however, more research is needed.

Despite these limitations, this study points out that the quality of life of children with AD is less impaired on school and social functioning compared to children with ADHD and ASD; although their quality of life is lower than typically developing children. Moreover, childhood AD are associated with less parental psychopathology and stress than children with ADHD and ASD. By selecting subjects based on diagnosis, we were able to examine impairments in daily life and family functioning specific to each diagnostic group. This information could help the community to better understand the impact of different diagnoses on children and their families, and could help clinicians adjust child interventions and parental guidance. Although dimensional measurement of psychopathology is becoming more popular (see DSM-5; [33]), treatment decisions and reimbursement are still mostly guided by categorically measured diagnoses rather than symptoms, which is why we chose diagnosis rather than symptoms as our selection criterion. It could be that parental factors are less associated with children with AD compared to children with neurobiological or externalizing disorders, and/or that having a child with AD is less severe compared to having a child with ADHD-C or ASD. Future research should disentangle the bidirectional relation between parent factors and children referred with mental disorders, comparing different mental disorders. Our findings may also suggest that parental factors need to be considered in child treatment (e.g., focus on parental stress management), and perhaps may need to be considered more in children with ADHD/ASD than in children with AD.

## Summary

Anxiety Disorders (AD) in children are associated with functional child and parental impairment; however, the severity of AD, compared to other common child mental disorders, is unclear. This study compared children referred with AD to children with ASD, ADHD-C, ADHD-I, and a control group on both child and parental factors. It was found that the quality of life of children with AD is less affected in school and social functioning than children with other clinical diagnoses; however, their quality of life is lower than typically developing children on all domains. In addition, parental psychopathology of children with AD was higher than typically developing children, but lower than children with ADHD-I. Interestingly, parental stress of parents of AD children did not differ from parents in the control group and parents of ADHD-I children, and was lower than that of parents of children with ASD and ADHD-C. The direction of the findings remains to be investigated, that is, childhood AD impacts parental functioning less, is less caused/maintained by parental functioning, or both. Parental factors may need to be considered more in treatment of children with ADHD/ASD than AD.
